# Genetic polymorphisms of nerve growth factor receptor *(NGFR) *and the risk of Alzheimer's disease

**DOI:** 10.1186/1477-5751-11-5

**Published:** 2012-01-12

**Authors:** Hui-Chi Cheng, Yu Sun, Liang-Chuan Lai, Shih-Yuan Chen, Wen-Chung Lee, Jen-Hau Chen, Ta-Fu Chen, Hua-Hsiang Chen, Li-Li Wen, Ping-Keung Yip, Yi-Min Chu, Wei J Chen, Yen-Ching Chen

**Affiliations:** 1 Institute of Epidemiology and Preventive Medicine, College of Public Health, National Taiwan University, Taipei, Taiwan; 2 Department of Neurology, En Chu Kong Hospital, New Taipei City, Taiwan; 3 Graduate Institute of Physiology, College of Medicine, National Taiwan University, Taipei, Taiwan; 4 Research Center for Genes, Environment and Human Health, Taipei, Taiwan; 5 Department of Public Health, College of Public Health, National Taiwan University, Taipei, Taiwan; 6 Department of Geriatrics and Gerontology, National Taiwan University Hospital, Taipei, Taiwan; 7 Department of Neurology, National Taiwan University Hospital, Taipei, Taiwan; 8 Department of Laboratory Medicine, En Chu Kong Hospital, New Taipei City, Taiwan; 9 Center of Neurological Medicine, Cardinal Tien's Hospital, New Taipei City, Taiwan; 10 Department of Laboratory Medicine, Cardinal Tien's Hospital, New Taipei City, Taiwan

**Keywords:** *NGFR*, Alzheimer's disease, htSNP, haplotype

## Abstract

**Background:**

Loss of basal forebrain cholinergic neurons is attributable to the proapoptotic signaling induced by nerve growth factor receptor (NGFR) and may link to Alzheimer's disease (AD) risk. Only one study has investigated the association between *NGFR *polymorphisms and the risk of AD in an Italian population. Type 2 diabetes mellitus (DM) may modify this association based on previous animal and epidemiologic studies.

**Methods:**

This was a case-control study in a Chinese population. A total of 264 AD patients were recruited from three teaching hospitals between 2007 to 2010; 389 controls were recruited from elderly health checkup and volunteers of the hospital during the same period of time. Five common (frequency≥5%) haplotype-tagging single nucleotide polymorphisms (htSNPs) were selected from *NGFR *to test the association between *NGFR *htSNPs and the risk of AD.

**Results:**

Variant *NGFR *rs734194 was significantly associated with a decreased risk of AD [GG vs. TT copies: adjusted odds ratio (OR) = 0.43, 95% confidence interval (CI) = 0.20-0.95]. Seven common haplotypes were identified. Minor haplotype GCGCG was significantly associated with a decreased risk of AD (2 vs. 0 copies: adjusted OR = 0.39, 95% CI = 0.17-0.91). Type 2 DM significantly modified the association between rs2072446, rs741072, and haplotype GCTTG and GTTCG on the risk of AD among *ApoE ε4 *non-carriers (*P_interaction _*< 0.05).

**Conclusion:**

Inherited polymorphisms of *NGFR *were associated with the risk of AD; results were not significant after correction for multiple tests. This association was further modified by the status of type 2 DM.

## Introduction

Dementia is a degenerative brain syndrome characterized by decline or loss in cognitive function [1]. About 30 million elders suffered from dementia worldwide in 2008 estimated by Alzheimer's Disease International. Alzheimer's disease (AD) is the most common causes of dementia and was the fifth leading cause of death for those aged 65 or older in the United States in 2006 [1]. In Taiwan, more than 160,000 people were demented in 2009 [2] and the number of AD patients keeps raising in many aging populations.

Degeneration of basal forebrain cholinergic neurons (BFCN) has shown to modulate cognitive function in AD patients [3,4]. Nerve growth factor receptor (NGFR, also called p75^NTR^) is one of the receptors of NGF and is expressed at the end of cholinergic axon [5,6]. The gene encoding *NGFR *is located on chromosome 17q21-q22. In normal brain, NGFR regulates tyrosine kinase receptor type 1 (TrkA), another receptor of NGF, and induces the signaling of neuronal cell survival [7,8]. In contrast, in AD brain, NGFR acts as a proapoptotic receptor in neuron cell death via binding to amyloid-beta (Aβ), NGF, or proNGF [9,10]. As a whole, NGFR plays multiple roles (survival and apoptosis) in human brain, dependent on the cellular context.

So far, only one study assessed the association between *NGFR *genetic polymorphisms and the risk of AD in an Italian population [11]. Cozza et al. found that variant rs2072446 was associated with a significantly decreased risk of familial AD [additive model: odds ratio (OR) = 0.28] [11]. However, no association was observed for other *NGFR *SNPs (rs741072, rs2072446, rs2072445, and rs734194) and the risk of sporadic AD [11]. In addition, type 2 DM has been related to the change of NGFR expression in rat brain [12] and cognitive impairment and dementia in the elderly [13-15]. However, no study has explored how type 2 DM affects the association of *NGFR *polymorphisms with the risk of AD.

NGFR plays an important role in neuronal survival and apoptosis, which may be related to AD pathogenesis. However, only one study explored the association between sequence variants of *NGFR *and AD in a Caucasian population. Therefore, we hypothesized that *NGFR *genetic polymorphisms were associated with the risk of AD in Chinese population. In addition, NGFR may involve in diabetic encephalopathy through neuronal apoptosis. Therefore, this study further investigated how type 2 DM modified the association of *NGFR *genetic polymorphisms with the risk of AD.

## Materials and methods

### Study Population

This was a case-control study. A total of 295 sporadic AD patients were recruited from neurology clinic of three teaching hospitals in northern Taiwan from 2007 to 2010. Healthy controls (n = 406) were recruited from elderly health checkup and volunteers during the same period of time. All participants were Chinese aged 60 years or older. Participants with the following diseases were excluded: hemorrhagic stroke, organic brain tumor, central nervous system diseases (e.g., Parkinson's disease), depression, cerebral infarction, and dementia other than AD (e.g., vascular dementia and mixed-type dementia, etc.). This study was approved by the institutional review board of each hospital and College of Public Health, National Taiwan University. Written informed consent was obtained from each study participant. The consent from the legal guardian/next of kin was obtained when patients had serious cognitive impairment.

A questionnaire was administered to collect information on demography, comorbidity (e.g., DM and stroke), life style (e.g., cigarette smoking, alcohol, tea or coffee consumption, and exercise); and family history. Blood sample was collected in a tube containing EDTA from each participant. Genomic DNA was extracted by using QuickGene-Mini80 kit (Fujifilm, Tokyo, Japan). After further exclusion of participants without blood samples, a total of sporadic 264 AD patients and 389 controls were included for data analysis.

### Dementia Evaluation

This study included only late-onset (age ≧ 60) non-familial AD. One neurologist at each hospital performed clinical examination to screen potential dementia cases. Mini-Mental State Examination (MMSE) [16] and Clinical Dementia Rating (CDR) [17] were used to access their cognitive function. The diagnosis of dementia was done by using Diagnostic and Statistical Manual of Mental Disorders, Fourth edition (DSM-IV) [18]. Head images, computed tomography and magnetic resonance imagings, were taken to exclude organic lesions. Diagnosis of AD was further determined by National Institute of Neurological and Communicative Diseases and Stroke - Alzheimer's Disease and Related Disorders Association (NINCDS-ADRDA) Alzheimer's Criteria [19]. Short Portable Mental Status Questionnaire [20] was used to assess cognitive function in controls to exclude participants with possible dementia and other mental disorders.

### Selection of Single Nucleotide Polymorphisms (SNP) and Genotyping Assay

Eleven common SNPs (frequency ≧ 5%) in *NGFR *were identified by using Han Chinese Beijing, China (CHB) genotype data from the International HapMap Project http://hapmap.ncbi.nlm.nih.gov/. Modified Gabriel et al. algorithm [21,22] was used to define haplotype block by using Haploview program http://www.broadinstitute.org/haploview/haploview. A total of 5 htSNPs (rs2072445, rs2072446, rs734194, rs741072, and rs741073) with an r^2 ^of 0.87 were selected in *NGFR *gene by tagSNP program [23] (Table 1). Five SNPs spanning *NGFR *formed one block.

**Table 1:** Characteristics of the study population

	Alzheimer's disease (n = 264)	Controls (n = 389)
	Mean ± SD	
Age (years)	79 ± 7	73 ± 6
	n (%)	
Female	172 (65%)	207 (53%)
Education		
Elementary	132 (50%)	40 (10%)
High school	93 (35%)	160 (41%)
College and above	39 (15%)	189 (49%)
Cigarette smoking		
Never	204 (77%)	321 (83%)
Former	42 (16%)	56 (14%)
Current	18 (7%)	12 (3%)
Alcohol consumption		
Never	230 (87%)	349 (90%)
Former	24 (9%)	13 (3%)
Current	10 (4%)	27 (7%)
Type 2 diabetes	48 (18%)	51 (13%)
Hypertension	103 (39%)	205 (53%)
Hyperlipidemia	48 (18%)	115 (30%)
*ApoE ε4 *carriers	107 (40%)	55 (14%)

*ApoE *denotes Apolipoprotein E gene.

Abbreviation: SD, standard deviation.

Genotypes of *NGFR *and Apolipoprotein E (*ApoE*) SNPs (rs429358 and rs7412) were determined by TaqMan^® ^Genomic Assays [24] using ABI 7900 HT fast real-time PCR system (Applied Biosystems, CA, USA). Genotyping success rate was greater than 95% for each SNP. Duplicate of 5% internal samples were selected for quality control purpose and the concordance rate reached 100% for each SNP.

### Statistical Analyses

Comparison of demographic characteristics between cases and controls were examined by using Student's t tests for normally-distributed continuous variables and chi-square tests for categorical variables.

Hardy-Weinberg equilibrium (HWE) test was performed for each SNP among controls to check genotyping error and selection bias. Partition-ligation-expectation-maximization algorithm was utilized to estimate haplotype frequencies by using tagSNP program [23].

To control for the confounding effect of age, frequency matching was used to match cases and controls on age within an interval of 5 years. The multivariate conditional logistic regression models were performed to estimate SNP- and haplotype-specific odds ratio (OR) and 95% confidence intervals (CI) for AD in participants carrying either 1 or 2 versus 0 copies of minor allele of each SNP and each multilocus haplotype. Potential confounders adjusted in the models included age, sex, and *ApoE ε4 *status. Stratified analyses were performed by *ApoE ε4 *status [25,26] and sex [27,28] because they have been related to the risk of AD. To control for type I error, the false discovery rate (FDR) and the single multiple-degree-of-freedom global test for the association between *NGFR *haplotypes and the risk of AD was performed. Given a significant global test, haplotype-specific tests can provide some guidance as to which variant(s) contribute to the significant global test.

Vascular risk factors (type 2 DM, hypertension, and hyperlipidemia) and *ApoE ε4 *status were known risk factors of dementia. Likelihood ratio test was used to evaluate the effect modification by each vascular risk factor on the association between *NGFR *polymorphisms and the risk of AD by comparing the model with main effects and interaction terms to the model with main effects only under the assumption of dominant model. Stratified analysis by type 2 DM status or *ApoE ε4 *status was performed to estimate OR for *NGFR *genetic polymorphisms and the risk of AD. All statistical tests were two-sided. SAS version 9.1 (SAS Institute, Cary, NC, USA) was used for statistical analyses.

## Results

### Characteristics of study population

A total of 264 AD cases and 389 controls were recruited in this study. As compared with controls, AD cases were older (79 vs. 72 years old), included more women (65% vs. 53%), had a lower education level (elementary school: 50 vs. 10 years), more with the history of type 2 DM (18% vs. 13%), fewer with the history of hypertension (39% vs. 53%) or hyperlipidemia (18% vs. 30%), and more were *ApoE ε4 *carriers (40% vs. 14%, Table 1).

### Haplotype-tagging SNPs in *NGFR*

**Figure 1:**
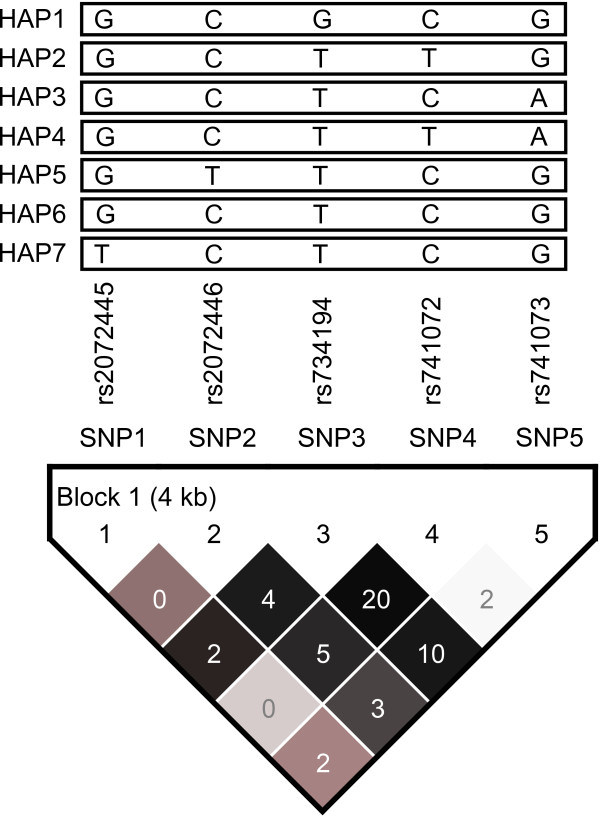
***NGFR *gene linkage disequilibrium (LD) plot**. The plot was generated by genotype data from this study by HaploView program. The level of pairwise D', which indicated the degree of linkage disequilibrium between two SNPs, was shown in the linkage disequilibrium structure in gray scale. The level of pairwise r^2^, which indicated the degree of correlation between two SNPs, was the number in the cell of the LD plot. Five common (frequency ≧ 5%) haplotypes were identified. Modified Gabriel et al. algorithm was used to define the haplotype block and 5 htSNPs formed one block.

Five htSNPs were selected from 11 common (frequency ≧ 5%) SNPs spanning *NGFR *formed one block, which was determined by the modified Gabriel et al. algorithm [21,22] (Figure 1). None of the NFGR SNPs was out of HWE (Table 2). The internal quality-control specimens did not show evidence of genotyping error as well. The minor allele frequencies (MAFs) of rs734194 (0.19 vs. 0.29) and rs741072 (0.41 vs. 0.30) were slightly different between HapMap data and our controls. Other SNPs (rs2072445 and rs2072446) showed similar frequencies.

**Table 2:** Characteristics of *NGFR *haplotype-tagging SNPs

	SNP1	SNP2	SNP3	SNP4	SNP5
	
	rs2072445	rs2072446 (Ser205Leu)	rs734194	rs741072	rs741073
Nucleotide change	G→T	C→T	T→G	C→T	G→A
Location	Intron	Exon	3'UTR	3'UTR	3'UTR
HapMap CHB					
MAF	0.09	0.15	0.19	0.41	0.25
Controls					
MAF	0.07	0.11	0.29	0.30	0.24
HWE *P value*	0.13	0.66	0.97	0.35	0.12
Cases					
MAF	0.08	0.11	0.25	0.34	0.25
HWE *P value*	0.17	0.72	0.57	0.21	0.37

Abbreviations: HWE, Hardy-Weinberg equilibrium; UTR, untranslated region; MAF, minor allele frequency; CHB, Han Chinese in Beijing.

### *NGFR *SNPs and AD risk

Variant rs734194 was significantly associated with a decreased risk of AD (GG vs. TT: OR = 0.43, 95% CI = 0.20-0.95) (Table 3). rs734194 remained significantly associated with an increased risk of AD under the assumption of additive model (OR = 0.71, 95% CI = 0.52-0.98, data not shown). After controlling for FDR, no significant association was observed for *NGFR *SNPs and the risk of AD.

**Table 3:** *NGFR *SNPs and the risk of Alzheimer's

	Co-dominant model							
	
SNP	0 copies		1 copy			2 copies		
			
	Case/control	OR	Case/control	OR (95% CI)	*p*	Case/control	OR (95% CI)	*p*
rs2072445	223/337	1.00	41/48	1.31 (0.76-2.28)	0.98	0/4	NA	
rs2072446	208/306	1.00	52/79	1.43 (0.86-2.37)	0.60	4/4	3.32 (0.65-16.85)	0.22
rs734194	146/196	1.00	103/160	0.79 (0.52-1.20)	0.48	15/33	**0.43 (0.20-0.95)**	0.06
rs741072	110/157	1.00	118/192	1.02 (0.67-1.56)	0.67	36/40	1.10 (0.59-2.06)	0.64
rs741073	145/222	1.00	105/151	1.04 (0.69-1.57)	0.26	14/16	2.04 (0.82-5.09)	0.13

All models were adjusted for age, sex, education, and *ApoE*ε*4 *status.

Abbreviations: OR, odds ratio; CI, confidence interval; NA, not applicable.

### *NGFR *haplotypes and AD risk

Seven common (frequency≥5%) haplotypes, composed by 5 htSNPs, were identified with a cumulative frequency of 97.3% in controls (Figure 1 & Table 4). Figure 1 demonstrated the LD structure by using the genotype data of controls in this study. The global *P *for the association between haplotypes and the risk of AD was 0.27. Participants carrying two copies of the minor Hap1 GCGCG had a significantly decreased risk of AD (OR = 0.39, 95% CI = 0.17-0.91). No haplotype was associated with AD risk under the assumption of additive model (data not shown). After correction for multiple tests by using FDR, significant association between *NGFR *polymorphisms and AD risk did not retain.

**Table 4:** *NGFR *haplotypes and the risk of Alzheimer's disease

Haplotype	Frequency among controls (%)	Co-dominant model							
		
		0 copies		1 copy			2 copies		
				
		Case/control	OR	Case/control	OR (95% CI)	*p*	Case/control	OR (95% CI)	*p*
Hap1: GCGCG	27.8	148/203	1.00	105/156	0.82 (0.54-1.25)	0.36	11/30	**0.39 (0.17-0.91)**	**0.03**
Hap2: GCTTG	21.9	159/234	1.00	90/140	0.94 (0.61-1.47)	0.79	15/15	1.01 (0.42-2.42)	0.99
Hap3: GCTTA	11.5	205/305	1.00	56/78	1.09 (0.65-1.84)	0.74	3/6	1.38 (0.27-6.97)	0.70
Hap4: GCTCA	11.3	197/304	1.00	65/81	1.37 (0.83-2.27)	0.22	2/4	0.94 (0.11-7.94)	0.95
Hap5: GTTCG	10.3	213/312	1.00	47/74	1.37 (0.81-2.33)	0.23	4/3	3.59 (0.66-19.42)	0.14
Hap6: GCTCG	8.9	217/323	1.00	46/63	0.97 (0.54-1.73)	0.92	1/3	0.18 (0.01-5.26)	0.32
Hap7: TCTCG	5.6	230/347	1.00	34/40	1.15 (0.63-2.12)	0.65	0/2	NA	

Global test *P *= 0.27									

Global test was testing for the null hypothesis that none of the haplotype was associated with AD risk.

Abbreviations: OR, odds ratio; CI, confidence interval; NA, not applicable.

### Effect of *ApoE ε4 *status

The *ApoE ε4 *carriers had a significantly increased risk of AD (OR = 4.45, 95% CI = 2.93-6.75) after adjusting for age and sex. The significant association remained after stratified by sex (male: OR = 3.45, 95% CI = 1.82-6.56; female: OR = 6.00, 95% CI = 3.38-10.62, data not shown).

### Effect modification by vascular risk factors

Among the vascular risk factors (hypertension, type 2 DM, and hyperlipidemia), type 2 DM was the only factor significantly modifying the association between *NGFR *polymorphisms (rs2072446, rs741072, Hap2, and Hap5) and the risk of AD. After stratification by type 2 DM status, significant associations were observed in some subgroups. Because *ApoE ε4 *status is an important risk factor for AD, we assess the effect modification by type 2 DM for *ApoE ε4 *carriers and non-carriers separately.

For *NGFR *SNPs, in *ApoE ε4 *non-carriers without type 2 DM, variant rs2072446 was associated with an increased risk of AD (TT+TC vs. CC: OR = 2.18, 95% CI = 1.19-4.00) (Table 5). In contrast, variant rs734194 was associated with a decreased risk of AD (OR = 0.28, 95% CI = 0.08-0.98, Table 5) among diabetic *ApoE ε4 *non-carriers. Significant interactions were observed between type 2 DM and *NGFR *rs2072446 and rs741072 on the risk of AD among *ApoE ε4 *non-carriers (*P*_interaction _= 0.007 and 0.04, Table 5). Except the interaction between type 2 DM and *NGFR *rs2072446 among *ApoE ε4 *non-carriers, other results were not significant after controlling for FDR.

**Table 5:** Effect modification by type 2 diabetes on the association between *NGFR *genotype and the risk of Alzheimer's disease stratified by *ApoE*ε*4 *status

	*NGFR non-*variant carriers		*NGFR *variant carriers		*P*_interaction_
			
	Case/control	OR	Case/control	OR (95%CI)	
***ApoE*ε*4 *non-carriers**					
rs2072445					
Diabetes	25/40	1.00	6/4	5.53 (0.53-57.61)	
Without diabetes	104/250	1.00	20/35	1.43 (0.70-2.92)	0.16
rs2072446					
Diabetes	26/26	1.00	5/18	0.48 (0.12-1.98)	
Without diabetes	89/227	1.00	35/58	**2.18 (1.19-4.00)**	0.007*
rs734194					
Diabetes	23/23	1.00	8/21	**0.28 (0.08-0.98)**	
Without diabetes	62/144	1.00	62/141	0.87 (0.52-1.45)	0.14
rs741072					
Diabetes	8/21	1.00	23/23	2.97 (0.91-9.74)	
Without diabetes	56/113	1.00	68/172	0.85 (0.50-1.43)	**0.04**
rs741073					
Diabetes	14/27	1.00	17/17	1.84 (0.57-5.89)	
Without diabetes	71/154	1.00	53/131	0.96 (0.57-1.61)	0.39

***ApoE ε4 *carriers**					
rs2072445					
Diabetes	12/5	1.00	4/1	NA	
Without diabetes	81/38	1.00	10/11	0.49 (0.16-1.46)	NA
rs2072446					
Diabetes	13/4	1.00	3/2	NA	
Without diabetes	79/44	1.00	12/5	1.49 (0.36-6.21)	NA
rs734194					
Diabetes	7/3	1.00	9/3	2.55 (0.20-33.18)	
Without diabetes	52/25	1.00	39/24	0.63 (0.28-1.46)	0.69
rs741072					
Diabetes	11/4	1.00	5/2	2.09 (0.15-29.98)	
Without diabetes	35/19	1.00	56/30	1.13 (0.47-2.69)	0.93
rs741073					
Diabetes	8/5	1.00	8/1	NA	
Without diabetes	51/31	1.00	40/18	1.24 (0.54-2.86)	NA

All models were adjusted for age, sex, and education.

Abbreviations: OR, odds ratio; CI, confidence interval; NA, not applicable.

*Result remained significant after controlling for FDR.

For *NGFR *haplotypes, among *ApoE ε4 *non-carriers, diabetic patients carrying minor Hap1 GCGCG had a decreased risk of AD (1 or 2 copies vs. 0 copies: OR = 0.28, 95% CI = 0.08-0.97, Table 6). In addition, among *ApoE ε4 *non-carriers, non-diabetic patients carrying minor Hap5 GTTCG were associated with a 2.12-fold increased risk of AD (95% CI = 1.13-3.99, Table 6). Among *ApoE ε4 *non-carriers, type 2 DM significantly modified the association of Hap2 and Hap5 with AD risk (*P*_interaction _= 0.04 and 0.01, Table 6). Results were not significant after controlling for FDR.

**Table 6:** Effect modification by type 2 diabetes on the association between *NGFR *haplotypes and the risk of Alzheimer's disease stratified by *ApoE*ε*4 *status

	*NGFR *variant non-carriers		*NGFR *variant carriers		*P*_interaction_
			
	Case/control	OR	Case/control	OR (95%CI)	
***ApoE*ε*4 *non-carriers**					
Hap1: GCGCG					
Diabetes	23/23	1.00	8/21	**0.28 (0.08-0.97)**	
Without diabetes	64/151	1.00	60/134	0.90 (0.53-1.51)	0.12
Hap2: GCTTG					
Diabetes	15/29	1.00	17/15	2.79 (0.83-9.43)	
Without diabetes	83/173	1.00	41/112	0.73 (0.42-1.26)	**0.04**
Hap3: GCTTA					
Diabetes	22/34	1.00	9/10	1.17 (0.30-4.51)	
Without diabetes	1.00	95/220	29/65	1.11 (0.57-2.14)	0.94
Hap4: GCTCA					
Diabetes	22/36	1.00	9/8	1.91 (0.42-8.78)	
Without diabetes	93/217	1.00	31/68	1.12 (0.59-2.11)	0.43
Hap5: GTTCG					
Diabetes	26/27	1.00	5/17	0.50 (0.12-2.09)	
Without diabetes	92/232	1.00	32/53	**2.12 (1.13-3.99)**	**0.01**
Hap6: GCTCG					
Diabetes	26/39	1.00	5/5	2.50 (0.32-19.61)	
Without diabetes	111/234	1.00	13/51	0.48 (0.21-1.10)	0.06
Hap7: TCTCG					
Diabetes	25/41	1.00	6/3	7.15 (0.55-93.39)	
Without diabetes	1.00	109/257	14/28	1.10 (0.48-2.54)	0.23

***ApoE*ε*4 *carriers**					
Hap1: GCGCG					
Diabetes	7/3	1.00	9/3	2.53 (0.19-33.57)	
Without diabetes	52/25	1.00	39/24	0.64 (0.28-1.48)	0.71
Hap2: GCTTG					
Diabetes	12/4	1.00	4/2	2.17 (0.13-35.20)	
Without diabetes	48/28	1.00	43/21	1.04 (0.42-2.57)	0.82
Hap3: GCTTA					
Diabetes	15/6	1.00	1/0	NA	
Without diabetes	72/40	1.00	19/9	1.43 (0.47-4.31)	NA
Hap4: GCTCA					
Diabetes	9/5	1.00	7/1	NA	
Without diabetes	70/41	1.00	21/8	1.49 (0.48-4.60)	NA
Hap5: GTTCG					
Diabetes	13/4	1.00	3/2	NA	
Without diabetes	80/44	1.00	11/5	1.48 (0.35-6.26)	NA
Hap6: GCTCG					
Diabetes	13/4	1.00	3/2	NA	
Without diabetes	66/41	1.00	25/8	2.42 (0.74-7.95)	NA
Hap7: TCTCG					
Diabetes	12/5	1.00	4/1	NA	
Without diabetes	82/39	1.00	9/10	0.53 (0.16-1.72)	NA

All models were adjusted for age, sex, and education.

Abbreviations: OR, odds ratio; CI, confidence interval; NA, not applicable.

## Disscusion

This is the first study exploring the association between *NGFR *polymorphisms and the risk of AD by using 5 htSNPs. We found that *NGFR *rs734194 was significantly associated with a decreased risk of AD, but this was not observed in the only previous study in an Italian population [11]. Possible reasons for the inconsistent findings between the Italian study [11] and ours include differences in sample size (Cozza et al. vs. ours, sporadic AD: 151 vs. 264, controls: 97 vs. 389), case selection (not available vs. incident cases), race (Italian vs. Chinese), study time period (not available vs. 2007 to 2010), mean age (AD: 65 vs. 79, controls: 64 vs. 73), and SNPs selected (4 functional SNPs vs. 5 htSNPs). This is also the only Asian study up to date. In addition, no significant association was observed for *NGFR *rs2072445 (in intron), rs2072446 (in exon), and rs741072 [in 3' untranslated region (UTR)], which is consistent with the findings of the Italian study [11]. rs741073 has not been explored for AD risk previously and was not associated with AD risk in our study. Although 5 htSNPs are in strong linkage disequilibrium (LD; i.e., high pairwise D' as shown in dark gray, Figure 1) and located within the same haplotype block, the pairwise correlation (r^2^) between rs734194 (SNP3) and any other SNP is quite low (SNP1:0.02, SNP2: 0.04, SNP4: 0.20, SNP5: 0.10, Figure 1).

Cholinergic hypothesis [3,4] has been used to elucidate the role of NGFR in AD pathogenesis because of selective loss of BFCN observed in AD patients. That is, elevated expression of NGFR and decreased TrkA may activate neuron apoptosis [29,30]. In addition, the binding of Aβ [31-33] and proNGF [9,34] to NGFR also induce neuron apoptosis. rs734194 is located on 3' UTR and thus plays an important role in regulating the mRNA stability and translational efficiency. Therefore, variations in rs734194 may reduce the expression of NGFR or the binding of NGF, Aβ, or proNGF to NGFR, which inactivates the neuron apoptotic signaling and leads to decreased risk of AD. It is also possible that the variations of rs734194 decrease the secretion of NGFR on BFCN and thus reduce the interaction of NGFR with Aβ and proNGF, which lower the neurotoxicity and apoptosis of BFCN. All together, these mechanisms may explain the protective effect of *NGFR *rs734194 on the risk of AD observed in this study.

We found that Hap1 GCGCG was significantly associated with a decreased risk of AD. rs734194 is the only SNP carrying the variant allele in Hap1. Therefore, the significant association of Hap1 and AD may be attributable to rs734194. It is also possible that other rare polymorphisms not analyzed here are responsible for the association observed. Our finding was not comparable to the Italian study [11] because fewer SNPs were selected and no significant association was observed for *NGFR *haplotypes in that study.

This study found that type 2 DM significantly modified the association between *NGFR *polymorphisms and the risk of AD in *ApoE ε4 *non-carriers. It is possible that type 2 DM modifies the association between *NGFR *and AD via the following mechanisms: (1) hyperglycemia [35-37], (2) altered insulin level and sensitivity in the brain [13,38-40], and (3) diabetes-related vascular diseases, e.g., hypertension and arterial disease [41]. In addition, *ApoE ε4 *status affects cholesterol metabolism and may act together with DM to modulate the risk of AD [42-44]. The significant effect modification was only observed in *ApoE ε4 *non-carriers, which may be due to the counteracting effect between *ApoE ε4 *allele (increase AD risk) and variant of *NGFR *(protective effect) on AD risk.

This study has some strengths. No study has investigated the role of *NGFR *polymorphisms on the risk of AD using a set of representative htSNPs and their corresponding haplotypes. In this study, 5 htSNPs were selected via a systematic approach and captured over 85% of genetic information in *NGFR *(estimated by tagSNP program). In contrast, the only prior study [11] assessed 4 *NGFR *SNPs, which capture only 14% of genetic information in the gene. Second, the sample size of our study is larger than the Italian study (Cozza et al. vs. ours, sporadic AD: 151 vs. 264, controls: 97 vs. 389). In addition, this study has over 90% power to detect an OR of 0.43 for the main effect and 78% power to detect an OR of 0.28 for the interaction between *NFGR *and type 2 DM on the risk of AD. Third, no study has assessed this association in Chinese population and identified *NGFR *SNPs representative for this ethnic group. Last, the use of brain image increased the validity of AD ascertainment and reduced misclassification of disease subtypes.

This study has some limitations. DM status was self-reported and thus may be biased. However, in our questionnaire, this information was further confirmed by asking if there was a previous diagnosis or taking medications for type 2 DM after seeing a doctor. Because DM is a major disease, participants' recall of DM diagnosis and their awareness of DM should be relatively accurate [45-47]. As a whole, the chance of recall bias was low.

In summary, this study found that *NGFR *htSNPs and haplotypes were associated with AD risk. Type 2 DM significantly modified the association between *NGFR *polymorphisms and the risk of AD in *ApoE ε4 *non-carriers. Although these findings did not reach statistical significance after correction for multiple tests, it is possible that the *NFGR *polymorphisms were associated with familial AD. This is because NGFR rs2072446 was associated with a decreased risk of familial AD in the Italian study [11]. Most of sporadic AD cases are *ApoE ε4 *non-carriers (60%) observed in this and other studies, therefore, our findings shed light on the importance of identifying genetic markers in *ApoE ε4 *non-carriers. Future large studies are warranted to confirm our findings.

## Competing interests

The authors declare that they have no competing interests.

## Authors' contributions

HCC: data analyses and manuscript writing; YS: participant recruitment; LCL: technical review; SYC: data analyses; WCL: technical review; JHC: participant recruitment and study design; TFC: participant recruitment; HHC: genotyping; LLW: biospecimen collection and treatment; PKY: participant recruitment; YMC: biospecimen collection and treatment; WJC: technical review; YCC: conceived of the study, genetic data analyses, manuscript writing, and project coordination. All authors have read and approved the final manuscript.
